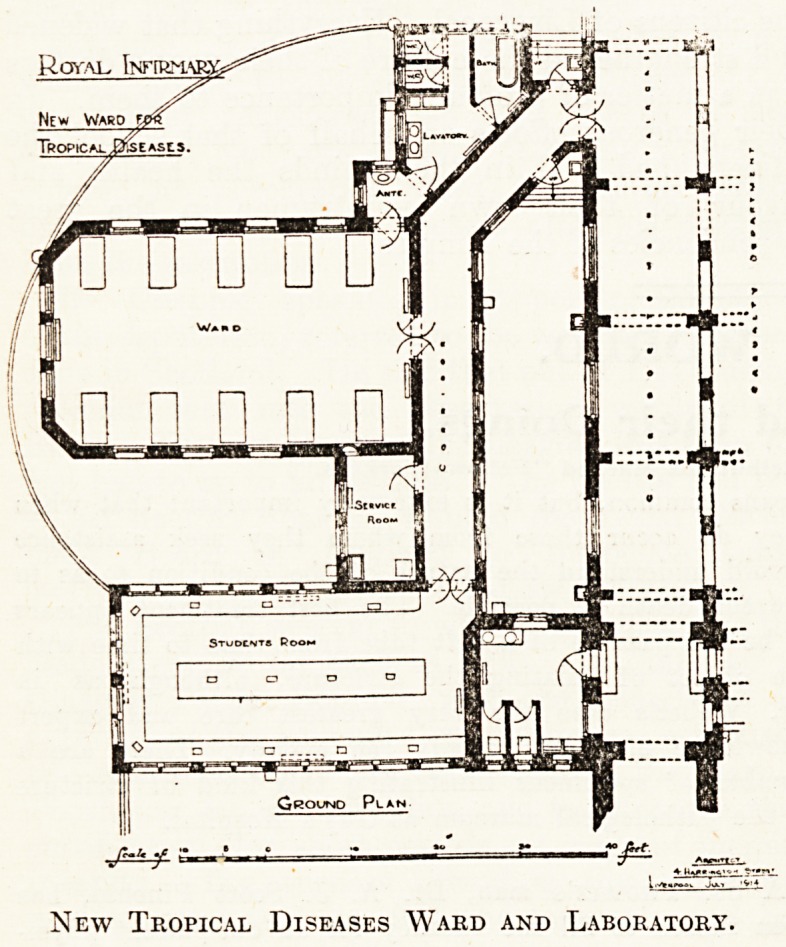# Opening of a New Ward and Laboratory for Tropical Diseases

**Published:** 1914-08-01

**Authors:** 


### THE LIVERPOOL ROYAL INFIRMARY.

### Opening of a New Ward and Laboratory for Tropical Diseases.

The late Mr. Alfred Jones, who did so much for the School of Tropical Medicine at Liverpool, left an adequate sum of money to build new laboratories and a specially designed new ward in the Royal Infirmary to be used exclusively by this school for the treatment of tropical diseases.

The Countess of Derby opened the Sir Alfred Jones Tropical Ward in memory of the founder of the Liverpool School on the 23rd ultimo.

### Description of the New Ward.

This ward, with its annex for students, is a one-storeyed building, so arranged as not to interfere with the access of light and air to the other wards of the hospital which are above it. The entrance is from the Royal Infirmary corridor leading to

New Tropical Diseases Ward and Laboratory.

the main staircase, in proximity to the casualty department on the other side of this corridor. The ward is 15 feet high, 25 feet wide, and 42 feet long, affording cubic contents of 1,575 feet to each of the ten beds contemplated. The laboratory for students—an apartment 40 feet by 20 feet—is placed on the north side, thus giving a suitable light for microscopical work, and there is a small serving room between it and the ward.

All the internal walls are faced with glazed bricks of quiet colour with coved angles at floor and ceiling, and the ward is floored in oak blocks, the other paving being in terrazzo, in keeping with the rest of the hospital. The outer walls are built of rustic brick with terra-cotta dressings, also in harmony with the main building. The heating is by low-pressure steam, with fresh-air inlets, and hopper-topped windows for ventilation. Electric lighting throughout. The building was designed by the late Mr. Francis Doyle, architect to the hospital, and the work has been completed by his brother, Mr. Sidney Doyle, and staff, with Messrs. Travis and Wevill as contractors.

We have had the privilege of inspecting this ward and laboratory, which are an addition to the teaching force of tropical medicine in Liverpool. Though awaiting their fittings, they promised to be excellent for their purpose in arrangement, in equipment, and generally. They showed an evident advance when contrasted with other accommodation provided for the study and treatment of tropical disease in the North. The Royal Southern Hospital, with great generosity and spontaneity, originally established a ward for the treatment of tropical diseases, and gave facilities for students which have been much appreciated. This accommodation was referred to in an article published in The Hospital of July 25, page 466. We have no doubt that the introduction of this new department to the teaching strength of the Royal Infirmary will secure that it shall prove in practice worthy of the noble traditions of medical efficiency which have made this institution so admittedly superior and exemplary in most of its arrangements.

When the Countess of Derby had inaugurated the new department she was presented with a souvenir in the form of a gold card-case of excellent workmanship and design. Mr. Danson invited the chairman, Mr. Wade Deacon, to accept on behalf of the Royal Infirmary the new ward from the School Committee, to be administered according to the agreement between these bodies. Mr. Wade Deacon, in accepting the gift, expressed the belief that the ward would bring renown to the infirmary, success to the men who studied there, and relief to the patients who might be treated in it. Subsequently Sir Thomas Barlow, President of the Royal College of Physicians, declared the Liverpool School of Tropical Medicine had long since passed the experimental stage. Its numerous expeditions carried to the very homes of death and disease had afforded records full of real actual romance. These expeditions, followed up by protracted researches in the school, had not only elicited valuable additions to the natural history of the diseases investigated, but had been justified up to the hilt by the actual improvement in the health conditions of the districts concerned owing to the enforcement of the lessons taught by research. The school, by the instruction given in tropical medicine and hygiene to graduates, many of whom had some tropical experience, was a great achievement. The scientific study of tropical diseases showed conclusively where and how the doctors should begin, and the particular measures which would most rapidly repay effort and expenditure. Already the school had passed through two stages, those of research and graduate teaching, and the proceedings that day inaugurated the commencement of the third stage, that of bringing a study of tropical disease within the curriculum of the medical undergraduates attached to the Liverpool School of Medicine. There was no higher scientific task than to cure a sick man, and the real justification for the step taken that day was the bringing of the laboratories and the sick man together so that there might be the readiest facilities to identify the real cause of the disease and to apply the quickest and most reliable methods of cure. One of the values of biological study was that it gave adequate mental drill as a preparation for the study of medicine. Bacteriology and parasitology had now reached that degree of thoroughness which could afford abundant opportunity, not only for the employment of observation and experiment, but for the reasoning faculty, and if they were to add bio-chemistry, then the educational potentiality was, if anything, still greater. He, therefore, submitted that for its pure educational value the more intimate association of tropical studies with the undergraduate stage could bring nothing but good. With the hospital stage pure and simple there were many reasons why it should find a place. So long as they maintained their colonies and their colonial medical service, and their kith and kin went out there to work, surely it was right to rouse the interest of their medical students in the fascinating problems which were crying for solution, which, when they were solved, would bring untold benefit to mankind. It was important to realise that the ordinary medical school curriculum was already heavily weighted. Some of them thought that the multiplication of classes took the men too much away from their ward work, and from the face-to-face acquaintance and familiarity with disease which made the real doctor. To that it might be answered that the tropical disease ward within the walls of that fine general hospital brought the immediate study of very interesting disease to the students' very door. He thought that the arrangement which would now exist was almost ideally complete. They had a constant succession of men of high scientific attainment bringing forth treasures new and old from their investigations far afield and from their laboratory researches. The new departure represented by that ward must have a beneficial bearing upon the citizens of Liverpool. Everything that widened and strengthened the culture of their young doctors was a matter of profound importance to them. In their generous efforts on behalf of that school the citizens had had in their minds the health and vigour of their own countrymen in the great dependencies of the Empire.

## Figures and Tables

**Figure f1:**